# The Role of Diagnostic Targeted Epidural Injection in the Diagnosis and Management of Chronic Spinal Pain: A Retrospective Analysis on 99 Patients

**DOI:** 10.5812/aapm-168719

**Published:** 2026-02-26

**Authors:** Alvise Martini, Vittorio Schweiger, Loris Anastasio, Luca Polati, Enrico Polati

**Affiliations:** 1 Department of Surgery, Pain Therapy Center, Verona University Hospital, Verona University, Policlinico GB Rossi, Verona, Italy

**Keywords:** Pain Management Practice, Chronic Spinal Pain, Spinal Pain Diagnosis, Targeted Epidural Injection

## Abstract

**Background:**

Chronic spinal pain, led by low back pain (LBP), which affects 619 million people globally and is projected to reach 843 million by 2050, is the main cause of disability. Although epidural steroid injections (ESIs) are recommended at Level I-A, their efficacy is often limited by technical factors, such as scarring, and they provide incomplete diagnostic information. We propose diagnostic targeted epidural injection (dTEI) via a Racz catheter, focused on detailed epidurography and anatomical exploration, as an essential diagnostic-therapeutic triage tool to overcome these drawbacks.

**Objectives:**

The objective of this study is to evaluate the efficacy of the procedure in a mixed low back pain population.

**Methods:**

A retrospective analysis was conducted on 99 patients with chronic radiculopathy, LBP, Post-Spine Surgery Syndrome type 2 (PSPS-2), or spinal stenosis (VAS > 40, duration > 3 months) treated with dTEI between January and December 2023. Outcomes, including Visual Analog Scale (VAS), Oswestry Disability Index (ODI), Quality of Life (QoL), and opioid use (morphine equivalent dose), were evaluated at one-month follow-up. Success was defined as either a VAS reduction greater than 50% or achievement of the Minimum Clinically Important Difference (MCID).

**Results:**

The mean age was 67.57 years. Significant improvements were observed in VAS scores (from 78.18 to 57.88, P < 0.001) and QoL scores (from 45.33 to 51.62, P = 0.001). Minimal therapeutic success according to MCID was achieved by 59.6% of patients. Opioid consumption was significantly reduced by 38%. Subgroup analysis showed that the spinal stenosis group achieved the best outcomes across all metrics. No correlation was found between the surgeon’s subjective assessment of technical success (contrast runoff/defect reduction) and superior clinical outcome.

**Conclusions:**

Diagnostic targeted epidural injection serves as a valuable diagnostic tool with significant therapeutic potential, optimizing patient selection in a stepwise management pathway. It effectively reduces pain and opioid use and provides detailed anatomical information to selectively guide nonresponders toward more complex and costly therapies, such as spinal cord stimulation or epiduroscopy. The primary value of the technique lies in diagnostic clarification and refinement of the therapeutic trajectory.

## 1. Background

Chronic low back pain (CLBP) remains the leading global cause of disability, affecting 619 million people in 2020. This burden is projected to reach 843 million cases by 2050, driven largely by population growth and aging (1). Often nonspecific in origin, CLBP is a complex condition influenced by interacting biophysical, psychological, and social factors rather than a single tissue injury. Identifying the pain generator and the most suitable therapeutic strategy can be difficult, even with the support of radiological and neurofunctional examinations, and inappropriate treatments may risk chronicizing disability and increasing the global burden rather than resolving the condition (2).

Epidural steroid injections (ESIs) are the most frequently used interventional first-line approach to CLBP (2). Their clinical efficacy remains debated, with multiple publications reporting conflicting conclusions. In 2025, a network meta-analysis concluded that ESIs offer little to no functional or pain relief advantage over placebo for chronic spine pain (3). Conversely, recent guidelines published by the American Society of Pain and Neuroscience and the American Society of Interventional Pain Physicians assessed the evidence for these procedures, assigning them a Level I-A recommendation and supporting their broad use (4, 5).

Although supported by scientific evidence, the use of ESIs presents 2 debatable aspects. First, these procedures often need to be repeated because their efficacy can be limited by technical factors, including incomplete drug diffusion due to anatomical distortion and epidural fibrosis after fixation. Second, even when performed under fluoroscopic guidance, the diagnostic information needed to identify the pain generator and plan more targeted interventions is often incomplete because of the lack of a complete epidurogram and missing information on levels superior and inferior to the injection site. To overcome these drawbacks, we used diagnostic targeted epidural injection (dTEI) via a Racz catheter as a first-line approach to CLBP and radicular pain.

As support for our view, a group from Egypt recently published a randomized controlled trial comparing a similar Racz catheter-based technique with conventional ESIs (6). We integrated dTEI into our diagnostic and therapeutic workflow with the aim of obtaining a detailed epidurogram, exploring epidural and foraminal anatomy, performing mechanical adhesiolysis where feasible, and administering steroids to selected and potentially multiple areas. This approach seeks to clarify epidural accessibility and anatomical feasibility for subsequent treatments, including pulsed radiofrequency (PRF), epiduroscopy, and spinal cord stimulation (SCS), especially in settings where MRI often fails to predict the degree of epidural fibrosis (7) and catheter-based exploration can offer essential real-time information.

Classical Racz epidurolysis is performed to mechanically and/or chemically remove adhesions resulting from inflammatory processes or previous spinal surgery, and its primary goal is therapeutic adhesiolysis (8). In contrast, the main goal of dTEI is diagnostic, focused on epidurography and triage, while providing potential therapeutic benefit in some patients. This distinction is crucial and differentiates our method from the classical Racz technique evaluated in randomized controlled trials and long-term evaluations (9 - 12).

## 2. Objectives

This study is a retrospective analysis based on real-world data, including patients treated with a modified dTEI technique according to standard clinical practice at our institution during 2023. We describe the outcomes related to pain relief, disability, medication use, and health-related quality of life in a cohort of 99 individuals with various pain etiologies treated with the dTEI procedure between January and December 2023. The data presented were collected as part of routine clinical practice at our unit.

## 3. Methods

### 3.1. Study Design

All patients who underwent dTEI from January to December 2023 were retrospectively analyzed. The indication for dTEI was established in cases of pain with VAS > 40 that was unresponsive to pharmacological and physical treatments. The pain had to have neuropathic or mixed characteristics, with a Douleur Neuropathique 4 questionnaire score of 4 or more (13), be localized in the lumbar spine and lower limbs, and persist for at least 3 months. Imaging of the lumbar spine had to exclude neoplastic disease or vertebral fractures, and patients could not have clear segmental motor deficits attributable to spinal pathology. The primary diagnosis had to fit one of the following categories: subacute or chronic radiculopathy, low back pain with radiculopathy (further named low back pain), PSPS-2, or spinal canal stenosis with neurogenic claudication.

Patient data were manually extracted retrospectively from our database, which was approved by the local ethics committee (reference 1751-CESC). The procedures performed were part of the usual treatment plan for patients with the described characteristics and were not part of a prospective study requiring dedicated institutional review board approval.

### 3.2. Diagnostic Targeted Epidural Injection Procedure

The patient was positioned prone with supportive padding to minimize lumbar lordosis. Twenty minutes before the procedure, antibiotic prophylaxis was administered. After sterile preparation, local anesthesia, and antibiotic prophylaxis, a 16G RX2 needle (Epimed, Dallas, TX, USA) was positioned through the sacral hiatus, followed by insertion of a pre-bent BREVI-XL 2 catheter (Epimed, Dallas, TX, USA).

An epidurogram was obtained by slowly injecting 2 - 6 mL of iodinated contrast (Omnipaque, GE Healthcare, Shanghai, China), according to patient tolerance, usually at the S1-L5 level in the midline. Based on the initial diagnosis and epidurogram findings, mechanical adhesiolysis was performed at any target level, and the presence of contrast runoff from the intervertebral foramina was evaluated by injecting 1 - 2 mL of iodinated contrast. Steroid medication (dexamethasone 8 mg in 10 mL saline solution) was infused throughout the procedure at clinically appropriate sites. Generally, a maximum of 10 mL of iodinated contrast and 10 mL of saline plus dexamethasone were sufficient to complete the procedure.

The physician performing the procedure documented a detailed description of the outcome, initial radiographic findings, and the presence or absence of contrast runoff at the treated segments in the operative report. The physician also stated whether the “surgical goal” had been achieved based on clinical and intraoperative findings and could suggest a possible next procedure if the treatment was ineffective. The “surgical goal” was subjectively defined and implied that the surgeon obtained runoff in the root suspected of causing radiculopathy or achieved opacification of an epidural area that was initially inaccessible to the contrast agent after mechanical dTEI.

### 3.3. Follow-up

All patients were scheduled for a one-month follow-up visit to assess the effectiveness of the procedure and decide on further treatment as part of usual care in our department. At both the preoperative visit and follow-up, patients completed the Oswestry Disability Index (ODI) (14) and EQ-5D-5L questionnaires, and VAS and Quality of Life (QoL) scores were collected (15). At follow-up, patients were also asked to rate their overall satisfaction with the procedure according to the Odom scale (1, excellent; 2, good; 3, fair; 4, poor).

At the one-month follow-up, based on the dTEI findings and the benefit achieved, 3 further interventions could be proposed to the patient: ganglion PRF for cases with good but short-lived benefits, especially if the symptoms were primarily radicular and there were no barriers to catheter positioning for PRF; epiduroscopy if it was not possible to reach the target because of epidural fibrosis that could not be addressed with mechanical dTEI alone; and SCS in cases of cul-de-sac fibrosis, which is frequently seen in patients with PSPS-2 and previous spinal instrumentation surgery.

### 3.4. Data Analysis

The primary diagnosis was classified into one of the following categories: subacute or chronic radiculopathy, low back pain with radiculopathy (further named low back pain), PSPS-2, and spinal canal stenosis with neurogenic claudication. Frequency distributions of demographic data were analyzed. Means of pre- and postoperative data were compared using paired t-tests, with statistical significance set at a two-tailed P-value < 0.05 for normally distributed data. Cohen’s d was calculated to quantify effect size (d = 0.2 - 0.5, small effect; d = 0.5 - 0.9, medium effect; d > 0.9, large effect). SPSS version 27 (IBM SPSS Statistics for MacOS, Version 27.0; IBM Corp., Armonk, NY, USA) was used.

The success of the procedure at one-month follow-up was defined in 2 ways: VAS reduction > 50%, considered fully satisfactory success; and minimal clinical improvement, defined according to the literature as reaching the Minimum Clinically Important Difference (MCID) for either VAS (> 15-point reduction) or ODI (> 10-point reduction) (16). Reductions in analgesic medication use after the procedure were considered, especially opioid medications converted into morphine equivalent doses. Outcome variables were analyzed in the general population, stratified by initial diagnosis, and according to whether the surgical goal was achieved based on the operator’s assessment, to evaluate whether a favorable outcome could be predicted at the end of the procedure.

## 4. Results

The analyzed sample included 99 patients: 36 males and 63 females, with a mean ± SD age of 67.57 ± 13.75 years. The main diagnoses included low back pain with radiculopathy (6.1%), PSPS-2 (13.1%), radiculopathy (74.7%), and spinal canal stenosis with claudication (6.1%). A total of 90 patients were using analgesic drugs preoperatively, of whom 42 were taking opioids (Table 1).

**Table 1.** Sample Description, Opioid Medication Use, Pre- and Post-operative Parameters ^a^

Variables	Values
**Pre-operative data**	
Patients	99
Gender	36 (36.4)
Age	67.57 ± 13.75
Drug therapy before surgery	90 (90.9)
Morphine consumption pre-op, mg	20.36 ± 27.86
Patients using opioids	42 (42.4)
**Diagnosis**	
Radiculopathy	74 (74.7)
Spinal stenosis	6 (6.1)
PSPS	13 (13.1)
Low back pain	6 (6.1)
**Post-operative data**	
Patients	99
Surgical goal achieved	65 (65.7)
**Satisfaction level**	
Grade 1 (excellent)	4.0
Grade 2 (good)	23.2
Grade 3 (fair)	36.4
Grade 4 (poor)	36.4
Following step drug therapy	77 (77.8)
Patients using opioids post-op	34 (34.3)
Morphine consumption post-op, mg	12.50 ± 14.91

^a^ Values are expressed as N (%) or mean ± SD unless otherwise indicated.

Regarding disability levels, the results indicated significant improvement after the procedure. The mean ± SD preoperative global ODI score was 43.41 ± 14.35, which decreased to 39.86 ± 16.82 (P = 0.014). Among patients in whom the surgical goal was achieved, as judged by the physician, the mean difference was slightly lower (3.20) and not statistically significant (P = 0.092) (Table 2).

The most marked improvement was observed in VAS pain scores. The preoperative mean ± SD was 78.18 ± 13.12, which decreased to 57.88 ± 24.04 postoperatively, with a mean difference of 20.30; this difference was highly significant (P < 0.001) and exceeded the MCID threshold. Similar results were observed in the subgroup of patients in whom the surgical goal was achieved, with a mean difference of 22.62 (P < 0.001).

The EQ-5D-5L index value showed a modest pre- to post-procedure change, with a preoperative mean ± SD of 0.43 ± 0.31 and a postoperative mean ± SD of 0.49 ± 0.33. The mean difference was -0.063, which was borderline significant (P = 0.049). Quality of life, measured with the QoL scale, showed significant improvement. The mean ± SD preoperative value was 45.33 ± 19.76, which increased to 51.62 ± 20.05 postoperatively, with a mean difference of 6.28 (P = 0.001). In patients for whom the surgical goal was achieved, a significant improvement of 4.65 points was observed (P = 0.046).

**Table 2.** Description of Outcome Variables in the Overall Population and in Subgroup Analyses Based on Opioid Use and Surgeon's Assessment ^a^

Groups	N	Pre-procedure	Post-procedure	P-Value	Cohen’s d
**General population**					
Index Value	99	0.43 ± 0.31	0.49 ± 0.33	0.049	0.19
ODI	99	43.41 ± 14.35	39.86 ± 16.82	0.014	0.22
VAS	99	78.18 ± 13.12	57.88 ± 24.04	< 0.001 ^b^	0.96 ^c^
QoL	99	45.33 ± 19.76	51.62 ± 20.05	0.001	0.31
**Only opioid users**					
Index Value	42	0.35 ± 0.35	0.40 ± 0.37	0.363	0.14
ODI	42	45.71 ± 14.45	43.48 ± 17.35	0.325	0.15
VAS	42	80.24 ± 12.59	63.33 ± 23.34	< 0.001 ^b^	0.77 ^c^
QoL	42	40.00 ± 18.18	47.62 ± 21.05	0.001	0.43
**Surgical goal achieved**					
Index Value	65	0.48 ± 0.30	0.51 ± 0.33	0.404	0.10
ODI	65	41.97 ± 14.41	38.77 ± 17.11	0.092	0.10
VAS	65	77.85 ± 13.74	55.23 ± 26.10	< 0.001 ^b^	0.96 ^c^
QoL	65	46.66 ± 19.43	51.31 ± 20.04	0.046	0.25

^a^ Values are expressed as mean ± SD.

^b^ ODI reduction thresholds > 10 and VAS > 15.

^c^ Cohen’s d values ≥ 0.5.

Analysis of clinical outcomes by diagnosis showed significant variations in the main outcome parameters (Index Value, ODI, VAS, and QoL) pre- and post-procedure (Table 3). VAS was the parameter that showed the most consistent and significant improvements across all diagnostic groups. QoL improved in all groups, with especially marked improvements in patients with PSPS and spinal stenosis. Disability measured by ODI showed less consistent changes, with significant improvement only in the spinal stenosis group. The Index Value showed moderate increases, significant only in the low back pain, PSPS, and spinal stenosis groups. The spinal stenosis group achieved the best outcomes in terms of pain reduction and improved disability and quality of life, with significant changes in all analyzed variables.

**Table 3.** Description of Outcome Variables in Subgroup Analyses Based on Initial Diagnosis ^a^

Groups	N	Pre-procedure	Post-procedure	P-Value	Cohen’s d
**Low Back Pain**					
Index Value	6	0.55 ± 0.10	0.59 ± 0.11	0.046	0.20
ODI	6	32.33 ± 15.31	35.67 ± 9.75	0.200	0.10
VAS	6	80.00 ± 17.89	50.00 ± 17.89	0.001 ^b^	0.80 ^c^
QoL	6	41.67 ± 19.15	50.00 ± 16.73	0.010	0.40
**PSPS**					
Index Value	13	0.31 ± 0.12	0.45 ± 0.15	0.050	0.30
ODI	13	46.92 ± 14.45	43.85 ± 15.57	0.180	0.15
VAS	13	79.23 ± 11.15	63.85 ± 15.57	0.005 ^b^	0.75 ^c^
QoL	13	36.15 ± 17.10	46.54 ± 18.42	0.020	0.50 ^c^
**Radiculopathy**					
Index Value	74	0.43 ± 0.31	0.48 ± 0.37	0.090	0.25
ODI	74	43.86 ± 14.87	39.16 ± 17.92	0.160	0.20
VAS	74	77.97 ± 13.14	57.57 ± 25.89	0.002 ^b^	0.70 ^c^
QoL	74	47.95 ± 20.34	53.72 ± 20.22	0.015	0.45
**Spinal Stenosis**					
Index Value	6	0.43 ± 0.30	0.52 ± 0.33	0.040	0.35
ODI	6	47.33 ± 7.55	38.00 ± 9.12	0.050	0.30
VAS	6	76.67 ± 15.05	56.67 ± 21.60	0.001 ^b^	0.85 ^c^
QoL	6	36.67 ± 10.80	46.67 ± 22.73	0.010	0.55 ^c^

^a^ Values are expressed as mean ± SD.

^b^ ODI reduction thresholds > 10 and VAS > 15.

^c^ Cohen’s d values ≥ 0.5.

Analgesic use followed a trend consistent with the outcome of the procedure. Among the 42 patients taking opioids, measured in morphine equivalents, average consumption decreased from 20.36 ± 27.86 mg to 12.50 ± 14.91 mg (P = 0.032). In this group, there was also a significant increase in QoL and a reduction in VAS from 80.24 ± 12.59 preoperatively to 63.33 ± 23.34 postoperatively (P < 0.001), and QoL increased from 40.00 ± 18.18 preoperatively to 47.62 ± 21.05 postoperatively (P = 0.001).

Minimal therapeutic success, defined as VAS reduction > 15 or ODI reduction > 10, was achieved in 59 patients (59.6%), while a VAS reduction > 50% was recorded in 15 patients (15.15%). Patient perception of the procedure’s outcome was not very positive. Only 27.2% rated the result as excellent or good.

**Figure 1.**
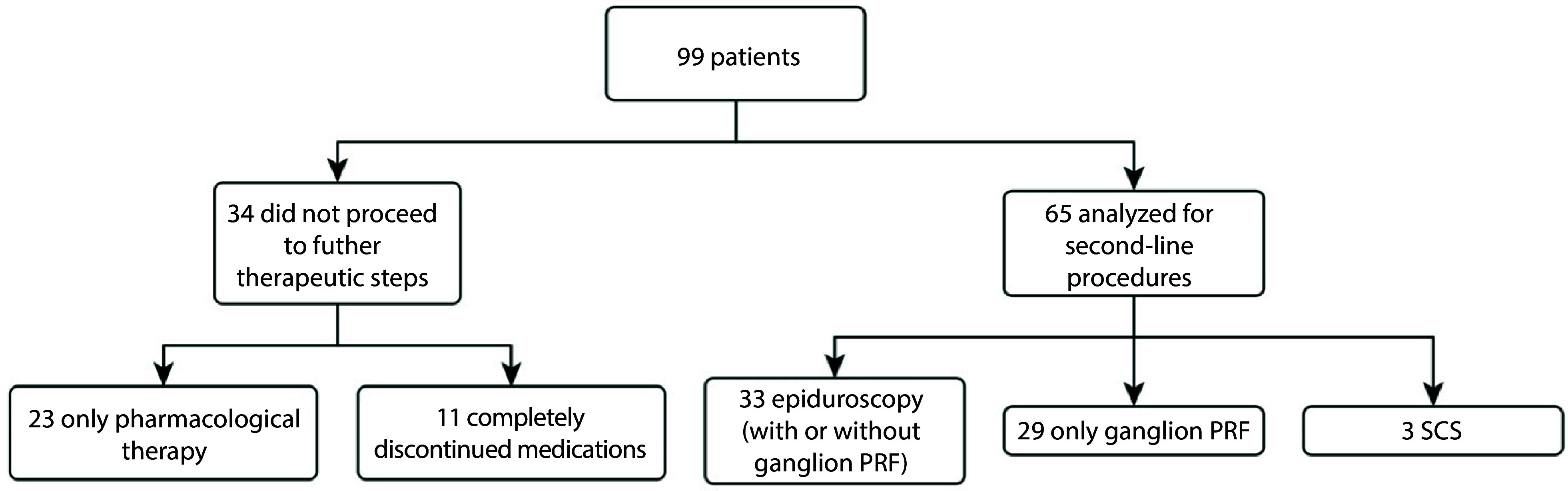
Flowchart of patient distribution and treatment paths at one-month follow-up

The execution of the procedure made it possible to guide patients with poor or transient outcomes toward a second intervention. Of the initial 99 patients, 34 did not proceed to further therapeutic steps at the one-month follow-up. Of these, 23 continued with pharmacological therapy only, while 11 completely discontinued medications. Therefore, the analysis of second-line procedures was conducted in the remaining 65 patients: 33 underwent epiduroscopy, with or without ganglion PRF; 29 underwent only ganglion PRF, without epiduroscopy; and 3 proceeded directly to SCS without further therapeutic steps (Figure 1).

## 5. Discussion

The success of interventional procedures for treating chronic lumbar and radicular pain is critically dependent on accurate patient selection. Despite advances in clinical evaluation, imaging, and neurophysiological studies, determining the most appropriate therapy for complex patients, particularly those with PSPS-2 and radicular symptoms, remains extremely challenging. Relying solely on imaging modalities such as MRI to assess epidural space accessibility for subsequent treatments, including PRF or epiduroscopy, can frequently lead to unsuccessful or impossible procedures. Bosscher and colleagues highlighted that conventional imaging often fails to predict the true anatomical condition of the epidural space, especially severe epidural fibrosis that may render the space inaccessible in instrumented PSPS-2 cases (7).

Our procedure is not primarily intended as a definitive resolution for all patients but functions as a powerful diagnostic and triage tool. As shown by our results, 15% of patients experienced a VAS reduction greater than 50% at one month, and 59.6% achieved clinically meaningful benefit according to MCID criteria (16). Although VAS is a unidimensional scale with inherent limitations, it showed a significant decrease in the general population and in the analyzed subgroups, with large Cohen’s d values, indicating a substantial effect of the procedure on reported pain.

We also observed statistically significant improvements in disability (ODI) and quality of life (EQ-5D-5L). In particular, EQ-5D-5L, a multidimensional and internationally recognized scale, showed a statistically significant improvement that exceeded the minimal clinically important difference estimated for CLBP, based on the minimum important change reported by Soer et al., although that estimate was based on the 3L version of the questionnaire (17). Another relevant point is that our baseline EQ-5D-5L value is among the lowest reported and may reflect the severity and chronicity typical of tertiary care referrals (18). These results should also be interpreted within the context of our analysis, which, although retrospective, used an intention-to-treat-like framework because no patients were excluded from the analysis.

A particularly noteworthy result is the significant 38% reduction in opioid consumption, with 19% of patients discontinuing opioid use entirely. These outcomes align with recent guidelines that prioritize nonpharmacological therapies and treatments capable of reducing reliance on potentially addictive substances for benign chronic pain (19).

The procedure’s critical function lies in its ability to selectively and effectively guide nonresponding patients toward more complex and costly therapeutic options. Some PSPS-2 patients benefit from epiduroscopy before eventual SCS (20, 21). However, in instrumented PSPS-2 cases, the epidural space may be inaccessible due to severe epidural fibrosis, which is often not detectable by MRI. A similar problem can occur when opting for ganglion radiofrequency in PSPS-2 patients with clinically well-defined radiculopathy, whereas ESIs may fail to provide complete information about the epidural space, pain generator, and feasible therapeutic options.

Diagnostic targeted epidural injection is not intended to be proposed as an alternative to Racz epidural adhesiolysis. Diagnostic targeted epidural injection is primarily conceived as a diagnostic and triage tool and may provide therapeutic benefit in selected patients, whereas Racz adhesiolysis is a therapeutic procedure supported by a robust body of literature (9 - 12). Nevertheless, the same catheter and needles are used for both techniques because of their versatility and proven structural reliability. As shown in this study, dTEI can also be therapeutic for a substantial number of patients, thereby reducing the need for more extensive and complex procedures.

Our analysis also revealed a counterintuitive finding that challenges conventional criteria for procedural success. Despite established literature emphasizing the importance of achieving contrast runoff through the foramen or reducing epidural filling defects as indicators of successful execution, our data showed a marked dissociation between this technical goal and clinical success. Patients in whom the technical objective was completed did not necessarily achieve better clinical outcomes. This discrepancy suggests that the etiology of radicular pain is more complex than simple mechanical foraminal obstruction.

The subgroup analysis of outcomes by diagnosis showed that the spinal stenosis group had statistically significant reductions in all outcome variables. This result is clinically relevant because it suggests that beginning treatment with dTEI may benefit these patients before clinicians pursue a more complex therapeutic pathway or await surgical intervention, when indicated. Overall patient satisfaction was not high. This discrepancy likely reflects a mismatch between patient expectations of therapeutic relief and the actual diagnostic purpose of the procedure.

Finally, the article by the Egyptian group, which was a randomized controlled trial analyzing ESIs versus a Racz catheter technique, seems to validate our rationale that percutaneous drug administration using a Racz catheter-based technique may be reasonable (6). Their patients achieved superior results compared with those in our cohort, which could be attributable to the use of a particulate steroid or to patient preselection, as they excluded patients in whom catheter advancement was impeded by dense epidural adhesions. In our real-world study, however, all patients were maintained in the analysis to reflect complete clinical practice. In line with our philosophy, we believe the utility of dTEI is to select patients for more complex procedures while simultaneously offering effective treatment for pain sustained by less complex clinical conditions.

### 5.1. Limitations

This study has several limitations. First, its retrospective design and lack of a control group limit the ability to establish a definitive causal relationship between the intervention and clinical outcomes. However, we believe that the value of our work lies in offering a complete analysis of real-world data, which provides clinical information and serves as an essential basis for planning future prospective studies. Second, the follow-up period was limited to one month, which may not fully capture the long-term efficacy or durability of treatment effects, although this was not the primary aim of the study because most patients subsequently underwent a second procedure after dTEI. Finally, the relatively small sample sizes in the subgroup analyses may have reduced the statistical power to detect between-group differences.

Despite these limitations, although the individual technical components are not novel, their systematic use as a first-line diagnostic approach in an unselected real-world population may represent a relevant contribution.

### 5.2. Conclusions

Diagnostic targeted epidural injection represents a valuable diagnostic tool with meaningful therapeutic potential. It fits optimally within a stepwise management pathway for chronic spinal pain, allowing clinicians to evaluate epidural accessibility, refine patient selection, reduce unnecessary procedures, and effectively guide subsequent treatments such as PRF, epiduroscopy, or SCS. Although short-term improvements in pain and quality of life were observed, the primary value of the technique lies in its diagnostic capability rather than in replacing classical chemical adhesiolysis validated in randomized trials. Prospective studies are warranted to confirm these findings and further clarify the relationship between epidurographic patterns, endoscopic anatomy, and clinical outcomes.

## Data Availability

The dataset presented in the study is available on request from the corresponding author during submission or after publication. The data are not publicly available due to privacy policy.
